# Supplementary material for: Regrowing the growth zone: metamorphosis kickstarts regeneration in the annelid Capitella teleta
Source: Development. 2026 Feb 11;153(3):dev204995. doi: 10.1242/dev.204995 (PMC12951290; doi:10.1242/dev.204995)
Supplement: Supplementary information [file develop-153-204995-s1.pdf]

Table S1. Ratio of ciliated hindgut: whole body length for individual animals in each experimental group measured.

3 day Uncut Juv	3 day JAL	3 day JAJ	Uncut st 9 larvae	3 day larvae	7 day Uncut Juv	7 day JAL	5 day JAJ	5 day larvae	14 day Uncut	14 day JAL	3 day Uncut Juv
0.445	0.372	0.353	0.310	0.245	0.490	0.163	0.269	0.268	0.463	0.537	0.445
0.522	0.456	0.078	0.353	0.198	0.501	0.200	0.241	0.191	0.451	0.478	0.522
0.396	0.273	0.084	0.319	0.193	0.441	0.272	0.385	0.278	0.399	0.516	0.396
0.395	0.382	0.159	0.308	0.000	0.383	0.353	0.251	0.170	0.357	0.633	0.395
0.442	0.534	0.056	0.258	0.092	0.432	0.365	0.349	0.233	0.402	0.414	0.442
0.595	0.370	0.197	0.305	0.000	0.336	0.348	0.201	0.148	0.383	0.553	0.595
0.429	0.483	0.267	0.330	0.118	0.518	0.188	0.086	0.105	0.377	0.308	0.429
0.379	0.448	0.230	0.294	0.000	0.535	0.284	0.325	0.109	0.458	0.452	0.379
0.482	0.384	0.172	0.269	0.000	0.339	0.380	0.131	0.172	0.357	0.403	0.482
0.364	0.370	0.222	0.254	0.000	0.414	0.353	0.524	0.208	0.427	0.272	0.364
0.432	0.365	0.453	0.258	0.000	0.593	0.330	0.109	0.191	0.350	0.565	0.432
0.433	0.407	0.262	0.254	0.000	0.464	0.360	0.344	0.158	0.375	0.276	0.433
0.278	0.249	0.258	0.310	0.000	0.527	0.311	0.363	0.111	0.379	0.338	0.278
0.460	0.459	0.617	0.224	0.076	0.413	0.239	0.239	0.176	0.259	0.435	0.460
0.539	0.383	0.277	0.222	0.000	0.556	0.401	0.218	N/A	0.392	0.569	0.539
N/A	N/A	N/A	0.189	0.087	0.586	0.407	N/A	N/A	N/A	N/A	N/A
N/A	N/A	N/A	0.350	N/A	N/A	0.358	N/A	N/A	N/A	N/A	N/A
N/A	N/A	N/A	0.326	N/A	N/A	0.282	N/A	N/A	N/A	N/A	N/A
N/A	N/A	N/A	N/A	N/A	N/A	0.379	N/A	N/A	N/A	N/A	N/A
N/A	N/A	N/A	N/A	N/A	N/A	0.321	N/A	N/A	N/A	N/A	N/A
N/A	N/A	N/A	N/A	N/A	N/A	0.281	N/A	N/A	N/A	N/A	N/A
N/A	N/A	N/A	N/A	N/A	N/A	0.268	N/A	N/A	N/A	N/A	N/A

Development • Supplementary information
